# Supplementary material for: Towards universal health coverage: The level and determinants of enrollment in the Community-Based Health Insurance (CBHI) scheme in Ethiopia: A systematic review and meta-analysis
Source: PLoS One. 2022 Aug 18;17(8):e0272959. doi: 10.1371/journal.pone.0272959 (PMC9387799; doi:10.1371/journal.pone.0272959)
Supplement: S2 File — (DOCX) [file pone.0272959.s002.docx]

**S2File: Examples of searching strategy for systematic review and meta-analysis on enrolment in Community based health insurance and its determinants in ethiopia, 2022**

| **Database** | **Example of searching strategy** | **Number of studies** |
| --- | --- | --- |
| PubMed | (("Enrolment" OR "enrol*" OR "Adopt*" OR "Uptake" OR "Uptak*" OR "using" OR "usage" OR "Utiliz*") AND (((((("Community-Based Health Insurance"[Mesh]) OR ("Community Based Health Insurance"[Mesh]))) OR ("Community Health Insurance")) OR ("health insurance")) OR ("Universal Health Insurance"[Mesh]))) AND (ethiopia) | 169 |
| Google Scholar | allintitle: Ethiopia enrolment OR Utilization OR uptake OR use OR join OR membership "community based health insurance" | 79 |
| Science Direct | ("enrolment" OR "Utilization" ) AND"(Community Based health insurance" OR "health insurance") AND ("Ethiopia)"(Research articles highlighted) | 171 |
| Scopus | TITLE-ABS-KEY ( ''enrolment AND Community based health insurance '' AND Ethiopia | 68 |
| Web of science | TOPIC: (Community based health insurance and Ethiopia) | 49 |
| Cochrane Library | ("Community based health insurance [MeSH]" OR "Health Insurance) AND Ethiopia | 22 |
