# Supplementary material for: Towards universal health coverage: The level and determinants of enrollment in the Community-Based Health Insurance (CBHI) scheme in Ethiopia: A systematic review and meta-analysis
Source: PLoS One. 2022 Aug 18;17(8):e0272959. doi: 10.1371/journal.pone.0272959 (PMC9387799; doi:10.1371/journal.pone.0272959)
Supplement: S3 File — (DOCX) [file pone.0272959.s003.docx]

**Table1:** JBI Critical Appraisal Checklist for prevalence studies used for assessing the individual quality of 11 crossectional studies included in the systematic review and meta-analysis, 2022.

| Authors, Year | The sampling frame appropriate to address the target population | The study subjects and the setting described in detail | Study participants sampled in an appropriate way | study subjects and the setting described in detail | Data analysis conducted with sufficient coverage of the identified sample | Valid methods used for the identification of the condition | Reliability of the instrument used to measure the condition | Appropriateness of Statistical analysis methods | Adequacy and management of Response rate | Total | Risk of bias |
| --- | --- | --- | --- | --- | --- | --- | --- | --- | --- | --- | --- |
| Geferso et al., 2022 | 0 | 0 | 0 | 0 | 0 | 1 | 1 | 0 | 0 | 2 | Low |
| Glagn et al., 2021 | 0 | 0 | 0 | 0 | 0 | 1 | 1 | 0 | 0 | 2 | Low |
| Tadesse, 2021 | 0 | 0 | 0 | 0 | 0 | 1 | 1 | 1 | 0 | 3 | Moderate |
| Nageso et al., 2020 | 0 | 0 | 0 | 0 | 0 | 0 | 0 | 0 | 0 | 2 | Moderate |
| Bifato et al., 2020 | 0 | 0 | 0 | 0 | 0 | 1 | 1 | 0 | 0 | 2 | Low |
| Alebel et al., 2020 | 0 | 0 | 0 | 0 | 0 | 1 | 1 | 0 | 0 | 2 | Low |
| Mekuria et al., 2020 | 0 | 0 | 0 | 0 | 0 | 1 | 1 | 0 | 0 | 2 | Low |
| Tsega et al., 2019 | 0 | 0 | 0 | 0 | 0 | 1 | 1 | 0 | 0 | 2 | Low |
| Belay et al., 2019 | 0 | 0 | 0 | 0 | 0 | 1 | 1 | 0 | 0 | 2 | Low |
| Workneh et al,2017 | 0 | 0 | 0 | 0 | 0 | 1 | 1 | 0 | 0 | 2 | Low |
| Shibeshi, 2017 | 0 | 0 | 0 | 0 | 0 | 0 | 0 | 0 | 0 | 2 | Moderate |

**Table2:** JBI Critical Appraisal Checklist for case control studies used for assessing the individual quality of 4 case control studies included in the systematic review and meta-analysis, 2022.

| Authors, Year | Were the groups comparable other than the presence of disease in cases or the absence of disease in controls? | Were cases and controls matched appropriately? | Were the same criteria used for identification of cases and controls? | Was exposure measured in a standard, valid and reliable way? | Was exposure measured in the same way for cases and controls? | Were the confounding factors identified? | Were strategies to deal with confounding factors stated? | Were outcomes assessed in a standard, valid and reliable way for cases and controls? | Was the exposure period of interest long enough to be meaningful? | Was appropriate statistical analysis used?  Total | Total | Risk of bias |
| --- | --- | --- | --- | --- | --- | --- | --- | --- | --- | --- | --- | --- |
| Meseret, et al., 2021 | 0 | 0 | 0 | 0 | 0 | 1 | 1 | 0 | 0 | 0 | 2 | low |
| Elmi et al, 2021 | 0 | 1 | 0 | 0 | 0 | 1 | 1 | 0 | 0 | 0 | 3 | Moderate |
| Getasew et al., 2020 | 0 | 0 | 0 | 0 | 0 | 0 | 0 | 0 | 0 | 0 | 0 | Low |
| Muluken et al., 2020 | 0 | 0 | 0 | 0 | 0 | 1 | 1 | 0 | 0 | 0 | 2 | Low |
