# Supplementary material for: Towards universal health coverage: The level and determinants of enrollment in the Community-Based Health Insurance (CBHI) scheme in Ethiopia: A systematic review and meta-analysis
Source: PLoS One. 2022 Aug 18;17(8):e0272959. doi: 10.1371/journal.pone.0272959 (PMC9387799; doi:10.1371/journal.pone.0272959)
Supplement: Attachment — Submitted filename: Response to Reviewers.docx [file pone.0272959.s007.docx]

**AUTHORS’ RESPONSE TO ACADEMIC EDITOR AND REVIEWERS**

1. **Author’s Response To Academic Editor**

**Dear: Gopal Ashish Sharma, MBBS, MD (Academic Editor)**

We thank you for a thorough reading and constructive comments and suggestions on our manuscript and for the opportunity to revise and resubmit. We are pleased to submit the revised research article “**Towards Universal Health Coverage: The level and determinants of enrolment in the Community-Based Health Insurance (CBHI) Scheme in Ethiopia: A Systematic Review and Meta-analysis**” for your consideration in the special collection of Plos One. On the following pages, you will find our responses to the questions raised from your side and two reviewers' comments. On behalf of my co-authors, I thank you for your consideration of this resubmission. We appreciate your time and look forward to your response.

Sincerely,

Aklilu Habte(MPH)(corresponding author)

[**aklilihabte57@gmail.com**](mailto:aklilihabte57@gmail.com)

**Dear Dr. Habte,**

Thank you for submitting your manuscript to PLOS ONE. After careful consideration, we feel that it has merit but does not fully meet PLOS ONE’s publication criteria as it currently stands. Therefore, we invite you to submit a revised version of the manuscript that addresses the points raised during the review process.

**General comment and suggestion**

At the outset, I would like to congratulate authors for highlighting the issue of UHC and its determinants within the context of Ethiopia's CBHI. Simultaneously, I would like to specially acknowledge the contributions of both reviewers for submitting exceptional and exhaustive reviews. It would definitely add to the value of the contents. Henceforth, authors are humbly requested to kindly consider/relook/rebutt the various minor revision points as raised by the reviewers. This would further enhance the scientific validity of the manuscript and will definitely improve the quality of the study. Suggestions proposed by one reviewer about publication bias need revisit. Please look into grammatical and typo errors specifically before submitting revised version. Please have a relook through track changes word document or seek help of the journal supportive staff if needed.

I am looking forward for re-submission so that the findings can be disseminated to the scientific community at the earliest possible and feasible.

**Response:** Thank you for providing us with the opportunity to revise our work by incorporating all of the valuable comments and suggestions made by the reviewers. We are also grateful for the reviewers' insightful comments and suggestions, which we received all of them were beneficial for further improving the manuscript. In the "Response to Reviewers" section below, we endeavored to answer all constructive remarks and ideas raised by both reviewers.

**Journal Requirements:**

When submitting your revision, we need you to address these additional requirements.

Comment1: Please ensure that your manuscript meets PLOS ONE's style requirements, including those for file naming. The PLOS ONE style templates can be found at

<https://journals.plos.org/plosone/s/file?id=wjVg/PLOSOne_formatting_sample_main_body.pdf> and

<https://journals.plos.org/plosone/s/file?id=ba62/PLOSOne_formatting_sample_title_authors_affiliations.pdf>

**Response:** Thank you for your suggestion. We have submitted the manuscript as per the journal requirement.

**Comment2:** In your Data Availability statement, you have not specified where the minimal data set underlying the results described in your manuscript can be found. PLOS defines a study's minimal data set as the underlying data used to reach the conclusions drawn in the manuscript and any additional data required to replicate the reported study findings in their entirety. All PLOS journals require that the minimal data set be made fully available. For more information about our data policy, please see http://journals.plos.org/plosone/s/data-availability.

"Upon re-submitting your revised manuscript, please upload your study’s minimal underlying data set as either Supporting Information files or to a stable, public repository and include the relevant URLs, DOIs, or accession numbers within your revised cover letter. For a list of acceptable repositories, please see http://journals.plos.org/plosone/s/data-availability#loc-recommended-repositories. Any potentially identifying patient information must be fully anonymized.

**Response:** We appreciate your kind suggestion for reminding us to incorporate the minimal underlying data set that we have used for this systematic review and meta-analysis. As per suggestion, we included in the “supportive information” section of the “Revised manuscript with track changes” as “S5File”, Line 512, Page17

**Comment3:** Please ensure that you refer to Figure 10 in your text as, if accepted, production will need this reference to link the reader to the figure

**Response:** we got our mistake and we have corrected and highlighted it in Line342, Page12 of the “Revised manuscript with track changes”

**Comment4:** We note that this manuscript is a systematic review or meta-analysis; our author guidelines, therefore, require that you use PRISMA guidance to help improve reporting quality of this type of study. Please upload copies of the completed PRISMA checklist as Supporting Information with the file name “PRISMA checklist”.

**Response:** we already have added it during the initial submission of the manuscript in the “Supportive information” section as “S1File”. We kindly suggest you see Line505, Page17 of the “Revised manuscript with track changes”

**Comment5:** Please review your reference list to ensure that it is complete and correct. If you have cited papers that have been retracted, please include the rationale for doing so in the manuscript text, or remove these references and replace them with relevant current references. Any changes to the reference list should be mentioned in the rebuttal letter that accompanies your revised manuscript. If you need to cite a retracted article, indicate the article’s retracted status in the References list and also include a citation and full reference for the retraction notice.

**Response:** Thank you for recommending that we have checked the appropriateness of the references we used in the manuscript. We reviewed all of the references as per your suggestion, and none of them have retracted references.

_____________________________END________________________________________

THANK YOU!!!

**AUTHORS’ RESPONSE TO REVIEWERS**

**Dear: Reviewers**

We thank you for a thorough reading and constructive comments and suggestions on our manuscript and for the opportunity to revise and resubmit. We are pleased to submit the improved research article **“Towards Universal Health Coverage: The level and determinants of enrolment in the Community-Based Health Insurance (CBHI) Scheme in Ethiopia: A Systematic Review and Meta-analysis”** for your consideration in the special collection of Plos One. On the following pages, you will find our response to the reviewers’ comments. On behalf of my co-authors, I thank you for your consideration of this resubmission. We appreciate your time and look forward to our responses to the comments and suggestions raised from your side independently as “Authors’ response to Reviewer#1”, and “Authors’ response to Reviewer#2”.

Sincerely,

Aklilu H. (MPH)(corresponding author)

[**aklilihabte57@gmail.com**](mailto:aklilihabte57@gmail.com)

**Authors’ Response to Reviewer**#**1**

**General comments**

I read the manuscript completely and I found that the authors have done remarkable research on the level and determinants of enrolment in the Community Based Health Insurance (CBHI) scheme in Ethiopia. I think this research could provide health policymakers with sound evidence to overcome the challenge of achieving Universal Health Coverage in the Ethiopian health system. Using a systematic review and meta-analysis method for answering the research question is a good option that could provide the needed evidence. Also, in this study, statistics, and other analyses are performed to a high technical standard and are described in sufficient detail. However, the manuscript needs minor improvements that could have a better presentation if they are considered by the authors.

**Response:** Thank you very much for taking the time to review our work and for your positive feedback. We got all your constructive comments and suggestions as valuable input for the readability of our work and we have tried to respond as follows. We give due emphasis to those grammatical and Punctuation errors by involving experts in language and literature and we have tried to correct them in the revised version of the manuscript.

**Specific comments**

**Comment1:** Google Scholar is a search engine and not a database. Please correct.

**Response:** Thank you for your comment. Of course, Google Scholar is not a database, but we used it as a search engine and thus list it as one of our tools for searching for relevant articles. However, we attempted to explain the distinction in the "Search strategy" section of the “Revised manuscript with track changes”, Line152-155, Page5.

**Comment2:** In the abstract section. It is best to draw a clearer picture of the study. If you started in a structured way, be sure to specify the other sections as well.

**Response:** We appreciate your insightful comment. We attempted to address your concern after an in-depth review of the contents in the “Abstract” section as per your suggestion but in line with the journal’s requirement.

**Comment3:** Try to make the introduction part of the abstract unique and not copy-paste from the manuscript.

**Response:** Thank you for the meticulous review of the manuscript. We have tried to re-synthesize most of the contents.

**Comment4:** Please use the following articles in this section of the introduction:

DOI: <https://doi.org/10.2147/CEOR.S254946>

DOI: <https://doi.org/10.1186/s12913-021-06673-0>

**Response:** thank you for your guidance. We explore those articles and we got valuable inputs that help us to strengthen the introduction section of our work. We used them as “Reference26 and 28” in both the “Introduction” and “Discussion” sections of the “Revised manuscript with track changes”.

**Comment5:** All abbreviations were defined when used for the first time in the manuscript: such as (LMIC)

**Response:** We appreciate your in-depth review of our work. As per suggestion we have added and highlighted them in the “Abbreviation” section of the “Revised manuscript with track changes”, Line488-489, Page16.

**__________________________________________________________________________________________________________END_______________________________________**

**THANK YOU!!!**

**Authors’ Response To Reviewer**#**2**

**General Comment:** Overall, this paper provides an interesting systematic review and meta-analysis that presents a pooled prevalence on the CBHI enrolment in Ethiopia. The answer to question 3 was provided as it is unclear if the study protocol was published on any platform, such as PROSPERO. Otherwise, all data seems to have been made available. In addition, the response to question 4 was given since the manuscript would benefit from further corrections as in the comments for consideration listed below and the attached Word document.

**Response:** Thank you very much for taking the time to review our work and for your positive feedback. We received your thoughtful, and generous review, along with helpful feedback and suggestions, as a valuable contribution to our ongoing work.

Of course, we didn’t register the study in PROSPERO and we accept it as a limitation. When we state this, we want to mention that we have checked to see if the study was registered by other authors, and we proceed to conduct the review process after assurance of the absence of duplication. Currently, there are difficulties in registering with PROSPERO at the moment as they are overloaded with COVID-19 reviews. Some researchers and findings were not big supporters of registration because it requires more effort and is often used to steal other people's work, and the benefit is primarily confined to avoiding duplication. Even in the journals that strongly endorse PROSPERO, they commonly mention that they recommend PROSPERO but do not regard it as a duty. Finally, we'd like to thank you for bringing it to our attention that we should think about registering, and we've decided to do so before commencing future SRMA.

We've attempted to respond to your comments and suggestions as follows.

**Response to Revision comments or further considerations:**

**Comment1.1:** Recommend some revisions to the introduction to facilitate readership and add clarity. Particularly, further clarification to the reader as to why certain countries were selected as a comparison in the first paragraph (lines 81-82)

Response: we got it is not the right way and we have corrected the statement by removing the figures for individual countries except the figure in Ethiopia. The amended statement was highlighted in the “Introduction” section of the “Revised manuscript with track changes”, Line76-79, Page3.

**Comment1.2:** why UHC would be assured through UHC, as stated in line 158. This notion reappears in lines 504-505, which implies that a country would solely require CBHI in order to reach UHC, which is quite a bold statement. The scope of the CBHI scheme can be quite limiting, not all-encompassing and can face sustainability issues, thus stating the prevalence of CBHI enrolment in this way seems reductionist. If this was not intended, then it could help to slightly modify these sentences, to not be too limiting.

**Response:** We appreciate your insightful and meticulous review of our work. When we see in the global context, the CBHI scheme is not the only mechanism to assure a UHC. When we came to the Ethiopian context, CBHI is the only working initiative on the way to achieving UHC. We have corrected some of the statements that seem reductionist in the “Introduction” section of the “Revised manuscript with track changes”, Line85-88, Page3. In addition in the “Discussion” section

**Comment2:** For the study design (lines 196-201), the study characteristics for PICOS are referred to in the S1File, i.e. the PRISMA guideline checklist. However, this is not clearly indicated within the methods section. Could this be clarified or incorporated?

Response: Thank you for your question and suggestion. as per your suggestion, we have tried to incorporate in the “Study selection and data extraction” section of the “Revised manuscript with track changes”, Line191, Page6.

**Commnet3:** Table 2 of the supplementary file 3 “S3File”, the critical appraisal tool, does not have any values within the tenth column. Was this intended or missed? Would recommend clarification.

**Response:** We appreciate your meticulous review of the manuscript. Now we have corrected and highlighted in “S3File” the “Revised manuscript with track changes”.

**Comment4:** In terms of the pooled prevalence, specifically line 456, it would be helpful to know what happened to the other four studies. Was no enrolment prevalence or number of people enrolled in the scheme reported? Would suggest describing this outcome for the four studies in the previous section called “Characteristics of included studies”. This way the reader does not have to examine the table and think of reasons why or be left guessing.

**Response:** Thank you for the in-depth review, and for giving us the chance to explain the confusing ideas in the manuscript. As our objective is to assess the level and identify the determinants of CBHI enrolment, we incorporate those epidemiological studies conducted with crossectional and case-control study designs. Of the total 15 studies, 11 were crossectional and the remaining four were case-control. Those four studies with no prevalence were case-control in design and they didn’t have a prevalence. We used 11 of 15 studies for estimating the pooled prevalence of CBHI enrolment. When we came to the determinants, we used all of the 15 studies. We hope we tried to answer your question to some extent.

**Comment5:** For Figure 4 (descriptions mentioned in lines 481-483), why does the grouping called “Since 2020” in the forest plot also include one study from 2020 (Alebel et al. 2020) and one from 2019 (Tsega et al. 2019), when those would actually make up the “Before 2020” category or grouping? Was this intentional?.

**Response:** Thank you for your meticulous review. Those displayed years with the author's name were publication years. But the forest plot was constructed based on the actual study period. There might be a possibility of a delay between the study period and publication year. The error that we have committed by misplacing one of the articles is now corrected in the revised version.

**Comment6:** For line 466, why in Figure 4 show a pooled estimate of 45.50% and a 95% confidence interval of 32.19 to 58.82 instead of the main reported finding?

**Response:** it was an error that we made during writing the output of the analysis. The right one is 45.5% and we have highlighted it in the “The pooled prevalence of CBHI enrolment in Ethiopia” section of the “Revised manuscript with track changes”, Line 272, Page 10.

**Comment7**: The manuscript briefly touches on stakeholders (e.g. lines 508-510 and 585-587), is there further information on the type and role of stakeholders involved in carrying the CBHI scheme forward and sustaining its implementation? It would be helpful to know more about the involvement and possible contribution of other stakeholders for the enrolment of the scheme.

**Response:** Thank you for asking. Of course, there are many stakeholders at different level who works on activities that enhance enrolment of the CBHI scheme and its management. Highly involved stakeholders are health institutions, health care providers including community health workers, international organizations(WHO), community and religious leaders, and non-governmental organizations (NGOs) like USAID. Their roles are community mobilization, training provision, some technical support with the financial management of the scheme, and logistic support like providing transportation materials for those who coordinated the CBHI scheme.

**Comment8:** The figures and tables require some standardization, especially in terms of the units and column headings, which either are not uniform or as clear as they could be. Would recommend another look to refine these.

**Response:** Thank you for your suggestion. We have prepared all the tables and figures as per the Plos One journal’s Manuscript submission guideline. The guideline recommends preparing the figures in the “Tif” file by renaming the captions as “Fig1, Fig2…”

**Comment9:** It seems that the study may indicate publication bias, as the lines 301-303 state: “Statistically, Begg's and Egger's tests were conducted, and a p-value < 0.05 indicated the presence of a small study effect (publication bias)” and the funnel plot (Figure 5) shows minor asymmetry towards the right side. If so, this would need to be reflected within the limitations section and the authors would need to discuss the implications of this publication bias.

**Response:** Thank you for giving us the chance to explain this critical question. As we stated in the “Heterogeneity and publication bias” section of the manuscript, publication bias was declared when the p-values of the Begg's and Egger's tests is <0.05. In this context, however, the p-values for Egger's and Begg's tests were 0.34 and 0.08, respectively, both were greater than 0.05, indicating that publication bias was less likely. Regarding the funnel plot, we tried to check it and reached the conclusion that publication bias is less likely, which was supported by the statistical findings of the Egger's and Begg's tests.

**Comment10:** The abstract is missing the heading “Conclusion”. Is the highlighted text within the attached Word document the conclusion?

**Response:** We have corrected it and highlighted it in the “Abstract” section of “Revised manuscript with track changes”, Line 62, Page 2.

**Comment11:** Minor edits to certain tables and figures are needed to improve consistency and comprehensibility to the reader. In addition, the supplementary files should also include figure titles. Supplementary Figure 1 has a few minor errors in the text, which would benefit from correction (e.g. “define” instead of “defined”, etc).

**Response:** Thank you for the comment. As per your suggestion, we have corrected all of the comments regarding the captions of figures and tables and highlighted them in the “Revised manuscript with track changes”

**Comment12:** Some of these formatting issues have been amended using tracked changes in the Word document. However, another look would be required prior to finalization to improve the English language and consistency of spelling throughout.

**Response:** We appreciate your detailed and insightful review of the manuscript. We found them all to be valuable and incorporated them into the revised manuscript. All of the issues outlined in the track changes were incorporated and highlighted throughout the revised manuscript.

**Comment13:** The references section requires another review to correct the inaccurately listed authors, specifically publications from organisations and agencies. Only some of these inaccurate references have been highlighted in the attached Word document.

**Response:** thank you for the in-depth review, which allows us to revise all the references that we have used throughout the work. As per your recommendation, we have corrected all the references accordingly and we cordially invited you to re-check them.

Comment14: Finally, was limiting the scope of eligible studies to English only an issue to consider in the limitations section of the discussion? Could any studies potentially have been missed?

**Response**: Thank you for your suggestion. We get it could be a limitation and we added it and highlighted it in the “Discussion” section of the “Revised manuscript with Track Changes”, Line 467, Page16.

Finally, we appreciate your thorough review of our work, as well as your insightful remarks and suggestions.

__________________________________________________________________________________________________________END________________________________________

THANK YOU!!!
